# Animal Models for Investigating Benign Essential Blepharospasm

**DOI:** 10.2174/157015913804999441

**Published:** 2013-01

**Authors:** Craig Evinger

**Affiliations:** Depts. of Neurobiology & Behavior and Ophthalmology, Stony Brook University, Stony Brook, NY 11794-5230

**Keywords:** Basal ganglia, blepharospasm, blink, cerebellum, motor adaptation, trigeminal.

## Abstract

The focal dystonia benign essential blepharospasm (BEB) affects as many as 40,000 individuals in the United States. This dystonia is characterized by trigeminal hyperexcitability, photophobia, and most disabling of the symptoms, involuntary spasms of lid closure that can produce functional blindness. Like many focal dystonias, BEB appears to develop from the interaction between a predisposing condition and an environmental trigger. The primary treatment for blepharospasm is to weaken the eyelid-closing orbicularis oculi muscle to reduce lid spasms. There are several animal models of blepharospasm that recreate the spasms of lid closure in order to investigate pharmacological treatments to prevent spasms of lid closure. One animal model attempts to mimic the predisposing condition and environmental trigger that give rise to BEB. This model indicates that abnormal interactions among trigeminal blink circuits, basal ganglia, and the cerebellum are the neural basis for BEB.

## INTRODUCTION

To create functional models of human movement disorders, the model organism should generate movements identical to those of humans. The blink system is ideal in this regard, because all mammals blink in the same way [1]. For all mammals studied, upper eyelid movements result from interactions among four forces. First, contraction of the phasic orbicularis oculi (OO) muscle actively closes the eyelid. The OO receives its input from the ipsilateral facial nerve [2]. Second, the tonically active levator palpebrae superioris (LP) muscle raises the eyelid and holds it open. Motoneurons in the oculomotor complex innervate the LP through the oculomotor nerve [3-6]. Third, Müller’s muscle, a smooth muscle that bridges the belly of the LP and its tendon, raises the eyelid. This muscle receives its innervation from the superior cervical ganglion [7-9]. Fourth, muscle and ligament attachments produce passive downward forces that oppose eyelid elevation [10-13]. Thus, the down phase of a blink occurs when the LP relaxes followed by a burst of OO activity. Combined with the passive downward forces, OO contraction rapidly lowers the eyelid. The LP resumes its tonic activity, following termination of OO activity, slowly raising the eyelid as the LP works against the continuously increasing passive downward force. The upper eyelid assumes its final position when the upward force of LP contraction matches the passive downward force.

Consistent with the common anatomical organization, the same physiological organization of blinking is similar among mammals. Stimulation of the supraorbital branch of the trigeminal nerve evokes two bursts of OO electromyographic activity, a short latency R1 and a longer latency R2. In humans, the longer latency R2 component produces most of the eyelid closure, [10] whereas the shorter latency R1 contributes most strongly to eyelid closure in non primate mammals [2, 14-17]. In all mammals investigated, blinks evoked by corneal stimulation elicit a single burst of OO activity [18-20]. Basal ganglia modulation of trigeminal blinks is identical for primates and rodents, and presumably the same for other mammals. The substantia nigra pars reticulata inhibits neurons in the superior colliculus that excite neurons in the nucleus raphe magnus. A serotonergic input from nucleus raphe magnus increases inhibition within spinal trigeminal blink circuits [15, 21-29]. Thus, the homologies in the anatomical organization and physiological control of the blink system among mammals make it ideal for developing animal models that closely mimic the eyelid focal dystonia benign essential blepharospasm (BEB). 

The characteristic signs and symptoms of BEB are spasms of eyelid closure, trigeminal hyperexcitability, excessive blinking, and photophobia. [30-34]. These characteristics are exaggerations of the normal blink adaptation to the corneal irritation created by dry eye [35]. Thus, it is not surprising that a significant proportion of patients report current or previous experiences of dry eye when first diagnosed with BEB [36-39]. Nevertheless, the vast majority of individuals with dry eye do not go on to develop BEB. The best explanation for this discrepancy is that BEB and other focal dystonias result from the confluence of a predisposing condition with an environmental trigger, the ‘two-hit hypothesis’ [31, 32, 40]. Dry eye or eye irritation is the strongest candidate to be the environmental trigger for BEB. The most likely predisposing factor for BEB and other dystonias is genetic. Although there is no clear genetic modification identified with BEB, there is compelling evidence that the gene responsible for the predisposing factor is autosomal dominant with low penetrance [41-47].

The most disturbing symptom for BEB patients is the involuntary spasms of eyelid closure that can produce functional blindness. Dystonic eyelid spasms are not tonic OO contractions, rather they are closely spaced bursts of OO activity, such that a new OO contraction begins before the eyelid has time to rise following the preceding burst of OO activity [31, 48, 49]. The primary treatments for these spasms are to reduce OO strength with botulinum toxin injections, [50-53] surgical removal of the OO muscle, [54] or killing muscle fibers with chemical agents [55]. One goal of animal models of blepharospasm is to create a system to test treatments that decrease OO contraction sufficiently to disrupt eyelid spasms without eliminating the blinking necessary to maintain the corneal tear film. 

One of the first models of involuntary eyelid closure was created by electrically stimulating premotor inputs to the facial nucleus of cats [56]. The investigators implanted stimulating electrodes into the facial nucleus, parabrachial region, red nucleus, interstitial nucleus of Cajal, the primary sensory nucleus of the trigeminal nerve, and into three reticular nuclei, ventral reticularis pontis oralis, reticularis parvocellularis, and reticularis centralis ventralis. In response to one long duration stimulus, only stimulation in the parabrachial region, the red nucleus and the interstitial nucleus of Cajal evoked a single, ipsilateral eyelid closure. The result for the red nucleus stimulation was somewhat surprising because the red nucleus projects primarily to contralateral OO motoneurons. [17, 57, 58-61] Ten to fifty Hz stimulus trains at these three sites produced sustained eyelid closure. Klemm *et al* (1993) [56] tested whether systemic drug treatment with atropine, haloperidol, molindone, diphenylhydramine or physostigmine reduced sustained eyelid closure in this model. None of the drugs modified electrically evoked eyelid closure even though the cats exhibited the general behavioral changes associated with the drugs. 

A possible role for 5-HT and catecholamines in blepharospasm was identified in reserpine treated rodents and rabbits. Acute reserpine treatment produces blepharospasm, muscle rigidity, akinesia, miosis, and ptosis [62-66]. Reserpine, RO4-1284, depletes 5-HT and catecholamine stores. Consequently, drugs that cause dopamine release, block monoamine oxidase, and/or activate 5-HT receptors tend to reverse the blepharospasm produced by reserpine treatment [67, 68]. The diverse pharmacological effects of reserpine, however, reduce its usefulness for identifying a pharmacological basis for eyelid spasms. Investigations focusing specifically on the role of 5-HT in eyelid spasms have been more informative. 

LeDoux and colleagues created another model of involuntary eyelid closure by microinjection of serotonin (5-HT) into the facial nucleus [69]. They reported that cats exhibited sustained eyelid closure ipsilateral to the 5-HT injection. This result was not a nonspecific effect of microinjection into the facial nucleus because microinjection of ketanserin, a 5-HT_2A_ and 5-HT_2C _antagonist, or saline did not cause sustained eyelid closure. LeDoux *et al* (1998) [69] demonstrated that pretreatment with oral administration of ritanserin, a 5-HT_2A_ and 5-HT_2C_ antagonist, diminished eyelid closure induced by 5-HT microinjection into the facial nucleus. The investigators linked these results to observations of successful treatment of some BEB patients with cyproheptadine, a H_1_ and 5-HT antagonist [70]. 

The blink system is exquisitely sensitive to central dopamine levels [71]. For spontaneous blinks, the blinks that occur without an external stimulus, systemic activation of dopamine receptors increases the blink rate and eliminating dopamine or blocking dopamine receptors reduces spontaneous blink rates [72-79]. Blinks evoked by stimulation of the cornea or periorbital region, trigeminal reflex blinks, respond oppositely to dopamine than do spontaneous blinks. Systemic treatment with L-dopa, apomorphine, a D_1_/D_2_ agonist, or enhancing dopamine release with nicotine reduces trigeminal reflex blink excitability [15, 80] and the speed of eyelid closure [81]. Conversely, the loss of dopamine neurons with Parkinson’s disease or experimental lesions in animals significantly increases the excitability of trigeminal reflex blinks [24, 28, 82, 83-90]. With this increased trigeminal excitability, touching the cornea can be sufficient to initiate a spasm of eyelid closure, reflex blepharospasm [82, 91-94]. These spasms of eyelid closure result from rapid bursts of OO contraction [24] as occurs in BEB eyelid spasms. Thus, dopamine depletion by itself causes reflex blepharospasm, but does not appear to be the direct cause of BEB as individuals with Parkinson’s disease do not exhibit spasms of eyelid closure in the absence of a blink evoking stimulus [95].

Despite the lack of convincing evidence that dopamine depletion alone causes BEB, abnormalities in dopamine transmission may be a proximate cause of the predisposing condition that allows the development of BEB. One genetic study reports polymorphisms in the DR5 gene associated with BEB, [46] although another investigation does not support this conclusion [41] and D5 knockout mice do not exhibit abnormal blinking [96]. Abnormalities in the D_2_ receptor, however, may set the stage for BEB. BEB patients show a decreased D_2_ binding in the striatum, [40, 97] and animal models of generalized and hemidystonia exhibit altered D_2_ binding [98, 99].

One rodent model of BEB mimicked the dystonia by creating a predisposing condition using minimal dopamine depletion and initiating an environmental trigger by creating a transient dry eye condition [100]. Rats received a unilateral, 6-OHDA lesion that caused a small loss of dopaminergic cells in the substantia nigra pars compacta. After creating this predisposing condition, a branch of the facial nerve providing approximately 30% of the input to the OO muscle was crushed near the OO. Because blinks made with the weakened OO inadequately reformed the tear film, the ensuing eye irritation served as the environmental trigger. This eye irritation initiated a series of trigeminal reflex blink modifications [35, 101, 102] that normally compensate for dry eye and eye irritation. In the presence of the predisposing condition, however, rats began to exhibit spasms of eyelid closure, excessive blinking, and trigeminal hyperexcitability. Even after OO reinnervation and concomitant resolution of the eye irritation, rats continued to exhibit these BEB-like blink abnormalities. Neither the predisposing condition nor the environmental trigger by themselves created these BEB characteristics. By itself, the small 6-OHDA lesion slightly increased trigeminal reflex blink excitability but did not generate reflex blepharospasm or spasms of eyelid closure. The OO weakening alone also increased trigeminal reflex blink excitability and resulted in the development of blink oscillations similar to those seen in human dry eye [35]. Combining the two conditions, however, produced eyelid abnormalities typical of BEB. Similar to this model, some individuals with facial palsy develop blepharospasm [103-105]. Consistent with the hypothesis that the predisposing condition enables maladaptive blink circuit adaptation in response to the eye irritation caused by inadequate lid closure, gold weight implantation to improve eyelid closure of the paretic eyelid reduced the drive for blink adaptation and the patient’s blepharospasm. 

This animal model [100] based on the two-hit hypothesis of focal dystonia is unlikely to have identified the ‘predisposing condition’ that allows BEB in humans. If dopamine loss was the predisposing condition in human BEB, a reasonable prediction is that BEB patients would be more likely to develop Parkinson’s disease than individuals without BEB. Such an increased incidence of Parkinson’s disease, however, does not appear to occur in BEB patients [106]. The eye irritation environmental trigger, however, is consistent with the human data [36-39] and the evidence that changes in the basal ganglia plays a role in the predisposing condition is compelling [40, 97-99]. Thus, the Schicatano *et al* (1997) model [100] suggests an outline for abnormal neural circuit interactions that support BEB.

The animal model of BEB indicates that the brain regions necessary to support BEB are trigeminal blink circuits, the basal ganglia, and the cerebellum. There is abundant evidence that adaptive modifications of blinking originate in trigeminal blink circuits [102, 106, 107]. Normally, the basal ganglia modulates inhibitory processes of trigeminal blink circuits to enhance or depress these adaptations. In pathological conditions, such as Parkinson’s disease, abnormal basal ganglia activity disrupts trigeminal blink circuits. [22-24] In the Schicatano *et al* (1997) animal model, [100] the predisposing condition distorts trigeminal blink circuit activity patterns so that maladaptive modifications occur in response to eye irritation [35]. The cerebellum is also critical in blink adaptation processes [101, 108, 109]. Trigeminal inputs to the cerebellum through mossy and climbing fibers [110-117] enable the cerebellum to support and maintain blink adaptations through indirect modulation of OO motoneuron depolarization and trigeminal system activity [68, 108, 117]. If the cerebellum receives abnormal trigeminal inputs from maladaptive learning processes, then the cerebellum will support and maintain this abnormal motor learning that originated in trigeminal blink circuits. Like previous studies indicating that the cerebellum is essential for the expression of dystonic movements, [118-120] the Schicatano *et al* (1997) rat model [100] predicts that the cerebellum is essential for maintaining spasms of eyelid closure created by abnormal trigeminal blink circuit motor learning enabled by a dysfunctional basal ganglia input This focus on abnormal motor learning as the proximate cause of BEB points to novel approaches to alleviate spasms of eyelid closure in BEB through modifying trigeminal motor learning.

An important goal of future animal models of BEB is to identify the predisposing condition and determine how it disrupts motor learning in trigeminal reflex blink circuits. The Schicatano *et al* (1997) model [100] uses a small dopamine depletion to create the predisposing condition, but evidence in humans [121] suggests that dopamine depletion is not the ‘predisposing condition’ in humans. The key to understanding how different predisposing factors, *e.g.*, genetic, dopamine loss, lead to BEB is to determine how basal ganglia dysfunction enables abnormal trigeminal blink circuit motor learning. One possibility is that different predisposing conditions may induce similar modifications in the pattern of basal ganglia activity. Parkinson’s disease causes an increase in the synchronicity of basal ganglia neuronal discharge and shifts the predominant frequency of this activity down to the beta range, 12 – 20 Hz [122-127]. Generalized dystonia also increases synchronicity of basal ganglia neurons, but the predominant frequency of this bursting pattern is 4 – 10 Hz, [126, 128-130] lower than that reported in Parkinson’s disease. This divergence in the frequency of basal ganglia outputs in Parkinson’s disease and dystonia can create dramatic functional differences in brainstem motor learning. Parkinson’s disease disrupts long term potentiation-like changes in blink amplitude, [131] whereas BEB enhances this form of motor learning [132]. Using animal models, it should be possible to identify modifications in the interconnected cerebellar and trigeminal blink circuits that modify motor learning when receiving abnormal patterns of basal ganglia activity.
